# On the need to better integrate the social environment in research on climate change and health: recommendations and thinking tools

**DOI:** 10.12688/openreseurope.17528.1

**Published:** 2024-05-28

**Authors:** Laurence Mabile, Lola Neufcourt, Matthew Chersich, Valériane Leroy, Cyrille Delpierre, Michelle Kelly-Irving

**Affiliations:** 1 CERPOP, Inserm, Université Toulouse III Paul Sabatier, Toulouse, 31000, France; 2 Wits RHI, University of the Witwatersrand, Johannesburg, 2000, South Africa

**Keywords:** Climate change, Planetary Health, One Health, EcoHealth, Social environment, Social determinants of health, Empirical studies

## Abstract

Social inequality impacts health, is aggravated by the consequences of climate change, and may be influenced by inappropriate policy responses. These interdependent effects create a self-perpetuating loop exacerbating the impact of climate dysregulation on health in an uncontrolled and poorly understood way. Holistic approaches to public health such as One Health, EcoHealth or Planetary Health are well suited to tackling the considerable and complex environmental and social issues underlying climate dysregulation. However, the extent to which research using such frameworks investigates social determinants of health is not clear. In this paper we discuss the ways in which the social environment has so far been considered in the literature to problematize and analyze the relationship between climate dysregulation and health within holistic frameworks and provide tools and recommendations to facilitate their apprehension. Social factors are investigated empirically only in a minor fraction of studies addressing the relation between climate and health in holistic frameworks, and not systematically. Barriers to such approaches are discussed. This work also provides two analytical tools (a process diagram and a knowledge framework) and a set of recommendations to help include the social environment more meaningfully in such frameworks. They are meant to facilitate our understanding of the current status of this type of research and to encourage trans-disciplinary and trans-sectorial endeavors towards directions which need to be taken to ensure societal factors and inequalities are placed at the center of research on climate and health and the ensuing policy response.

## Plain language summary

There is now a lot of scientific evidence showing that the social structures of human societies strongly influence our health and impact the consequences of climate change in many direct or indirect ways. As a result, some groups of people are likely to be more frequently exposed to unfavorable conditions and are potentially much more vulnerable than others. All these effects get trapped in a vicious loop that could dramatically aggravate in an uncontrolled way the effects of climate dysregulation on health, although the mechanisms and scale of this phenomenon are still poorly understood. In the context of the climate crisis, it is more important than ever to consider and understand social inequalities in research to make progress towards climate justice and health equity. This type of research is essential to help propose adequate mitigation/adaptation policies and interventions. In this paper, we review and discuss the ways in which the social environment has so far been considered in the research works that study the relation between climate change and health (health defined in a broad sense called holistic approach of health that considers health of all living organisms, ecosystems and of the planet as essential to human health). This article aims to facilitate the understanding of the current status of this type of research, and the directions which would need to be taken to ensure that social inequalities and societal factors are placed at the heart of climate and health research and of the ensuing policy response. It is part of the EU funded -ENBEL project whose main goal is to facilitate connections between researchers and policy makers in the fields of climate & health research.

## Introduction

Climate change is increasing the frequency and intensity of many extreme weather events, resulting in severe damage to the natural and social systems on which health depends ^1^. While mitigation strategies clearly need to be actively pursued, it is critical to base adaptation strategies on detailed knowledge of the numerous climate dysregulations, their impact, and their underlying interconnections with other major social and political challenges given the complexity of this global concern.

The devastating health and social consequences of such changes are part of a bigger global picture where the human and economic costs are already high. Climate change has adversely impacted health in many ways: from deaths, injuries, premature births and post-traumatic stress disorders caused by direct effects of extreme weather events, to a myriad of diseases due to indirect effects linked to air, soil, water, food quality and availability and to biodiversity loss ( WHO Meeting Report, 2015). Long neglected in climate change discourse, the climate change impact on health is now fully incorporated within the last Intergovernmental Panel on Climate Change (IPCC) assessment ^2^ which also highlights the interdependence of climate, biodiversity and populations, some of which more vulnerable than others.

Health is determined by our social conditions, resources and environment: the poorest people have the highest levels of morbidity and premature mortality ^3^. Furthermore, across the social spectrum, health follows a social gradient: on average the lower one’s socioeconomic position, the worse is one’s health ^4^. Social position also affects the other determinants of health, such as access and use of health services, psychosocial support, behavioral factors, education, food, housing, recreational activities and other societal resources, all critical for good health and well-being. These in turn are shaped by political, social, and economic forces that lead to inequity in a systematic way, produced by social norms, policies, and practices that tolerate or promote unfair distribution of and access to power, wealth, and other necessary social resources ^5^.

Climate change also meaningfully affects the social determinants of health which are critical in the construction of health ^6^. This happens in different ways, notably described in the WHO Commission on Social Determinants of Health final report and identified formally by Islam & Winkel ( UN Department of Economic & Social Affairs working paper, 2017), as affecting exposure, sensitivity and resilience of disadvantaged populations. These include increased exposure of poor populations to extreme heat, pollution and climatic disasters due to their residential hazardous location usually more prone to flooding, erosion, mudslides or close to waste sites, roads and factories; increased susceptibility of some population groups such as women, due to their typical daily tasks imposed by social norms, or some workers whose occupational location and activities, or populations living in poor quality housing or with comorbidities; and a decreased ability to cope with stressful events or to prevent or recover from physical and material damages (due to systematic resource-constraints such as lack of private resources, of insurance contracts, of access to public resources and services…) ^7, 8^. These driving forces can lead to property loss, reduced livelihoods, residential relocation, and other climate-related crises (infrastructure damages limiting access to healthcare, food insecurity, forced migration, war…). On top of this, social inequalities and per capita greenhouse gas emissions are positively correlated in some economic contexts, which implies that inequalities may even aggravate the climate change process ( UN Department of Economic & Social Affairs working paper, 2015). As poverty and social injustice continue to kill people on a grand scale, also gaining ground in high income countries ^9^, a vicious circle sets in that further amplifies climate change impacts on vulnerable populations, exacerbating climate impact on health in an uncontrolled vertiginous way that not only affects some populations disproportionately but also adds on the burden on medical services. According to the World Bank, the estimated number of people falling into extreme poverty due to climate change is ranged between 32 million and 132 million in most scenarios by 2030 ^10^. Therefore, it is critical to understand the global picture when to design adequate actions, not to imbalance the system or generate deleterious effects in other places.

As well as their own vulnerability to the effects of climate change, the most socially disadvantaged are also disproportionately burdened by new and often regressive policies which are set in place to foster behavioral or cultural changes ^11, 12^. Disadvantage caused by poverty, discrimination or geographical factors has led to the exclusion of some groups from digital technologies, sustainable foods or public transport infrastructures, areas which will be important to develop to deliver carbon neutral targets. For instance, when policy incentives are established to improve housing and facilitate shifts away from polluting vehicles, these must consider the challenges faced by people with low incomes and/or living in isolated rural places ^13^. As such, understanding and identifying the social patterning of the human health and wellbeing consequences of climate dysregulation and ecosystem disruption are key if we are to avail ourselves of actionable, reliable research and policy priorities ^14^. Thus, the climate emergency and social inequalities in health must be addressed simultaneously based on scientific actionable evidence to guide equitable and acceptable policy responses ^15^.

To tackle the variety of influential factors at stake, three holistic frameworks of health have gained prominence over the last decade, namely EcoHealth, One Health and more recently Planetary health. Among public health models, the holistic approaches aim to provide comprehensive frameworks to systematically investigate complex multifactorial relationships between political, social, cultural and environmental factors that underpin climate change impacts on health of humans, living organisms, natural ecosystems and of the planet. Although there are some minor differences, all three assume that humans and other animals share the same planet habitats and face the same environmental challenges, infectious agents as well as other aspects of health ^16^. The “ One Health” (OH) concept was introduced at the beginning of the 21st century, emphasizing that human and animal health are interdependent and connected to the ecosystems in which they exist. OH is also seen as « the collaborative effort of multiple health science professions, mainly medical and veterinary sciences and institutions—working locally, nationally, and globally—to attain optimal health for people, domestic animals, wildlife, plants, and our environment ». EcoHealth (EcoH) is described as being committed to fostering the health of humans, animals, and ecosystems (including microorganisms) and « to conducting research which recognizes the inextricable linkages between the health of all species and their environments ». Emphasis is placed on the threat that a resource-depleted, polluted, and socially unstable planet poses for health on the long run ^16^. Planetary health (PH) is the most recent ecological public health model in line with the 1970’s environmental and holistic health movements and with the indigenous knowledge of health. Compared to the WHO definition where “health is a state of complete physical, mental and social wellbeing and not merely the absence of disease or infirmity”, the definition of planetary health would be “the achievement of the highest attainable standard of health, wellbeing, and equity worldwide through judicious attention to the human systems—political, economic, and social—that shape the future of humanity and to the Earth’s natural systems that define the safe environmental limits within which humanity can flourish” ^5^.

Scientific papers are increasingly discussing the impacts of climate change on health through the lens of the above holistic frameworks. However, the extent to which social determinants of health have been investigated within such frameworks is not clear. In this paper, we discuss the ways in which the social environment is considered in problematizing and analyzing the relationships between climate dysregulation and health within holistic frameworks as well as the challenges that may be faced in such research works. We further provide some recommendations and tools that may facilitate our understanding of the current status of this research, and help foster research and actions towards directions which need to be taken to ensure that social inequalities and societal factors are placed at the center of research on climate and health and the ensuing policy response.

## Discussion

### Climate change, social environment & health within holistic frameworks: insights from the literature analysis

The climate change topic has been increasingly infiltrating research since the 1990s mainly after the first Intergovernmental Panel on Climate Change assessment was reported. In the life sciences and biomedical research literature, this topic represents though a very small proportion of health research in general and is significantly associated with holistic approaches of health (namely OH, EcoH, PH) that increasingly appeared in this literature over the past decade. These holistic models address a broader field of environmental investigation which make them well fitted to tackle environmental and social issues such as those conveyed by climate change. We carried out a literature analysis for which detailed information is available on the OSF repository [ https://osf.io/nmcva/?view_only=9940481c8b634e23b59ca327ee21e27f]. Among articles which simultaneously included i) the term “climate change”, ii) one of the holistic above-mentioned frameworks (OH, EcoH or PH) and iii) at least one term related to the broadly-defined social environment - i.e. related to any social organization, process, norm, habits, and characteristics possibly affecting health (directly or indirectly) including lifestyle behaviors -, we first found that a significant though limited number of articles have so far addressed the climate change topic through the lens of holistic approaches of health (Eco H, PH or OH; 595 in Wos, 393 in Pubmed). A fraction of these works further considered simultaneously some aspects of the social environment (57%; n=401) as follows.

The majority of such articles (80%) were not original research articles but theoretical works such as reviews or various types of commentaries, recommendations and method papers. Overall, the theoretical works covered a variety of knowledge and areas that pointed to challenges and problems faced across domains at a global level when working on holistic models of health. These areas were either addressed (i) through a theoretical lens such as syntheses on specific effects of climate change related to health; concepts development within the three holistic frameworks; rationales for their use in the context of environmental changes including climate change and critique of the different approaches; vulnerability rationales to climate and/or diseases (zoonotic mostly) and views on transforming the food system to better protect environment and health; or (ii) as practice papers such as lessons-learned from implemented approaches; public health/clinical practice & training considerations in such approaches; indicators of resilience monitoring; analyses of policies alignment to the Sustainable Development Goals and analyses of political agendas towards risk reduction.

Only a minority were empirical papers (20%), mainly observational, modelling, and assessment ones that globally not only addressed direct or indirect impacts of climate change on health but also tackled climate change and health as independent topics of education, training or risk monitoring. The major topic areas covered were related to public health & healthcare services, health professional practice & training, and nutrition. All aimed to provide new knowledge in domains often challenged at the human/animal or ecosystem interface, sometimes also considering planet resources.

With regard to the social factors studied in the empirical studies, a minority (19%) were treated as the main variable of interest / exposure / outcome, most of them were either treated as covariates (43%), often as descriptive variables or only discussed as having a possible influence (37%). None of the factors related to individual characteristics (gender, ethnicity, education) or to household assets (including occupation tenure and socio-economic items) were addressed as an exposure or outcome. Social determinants were more often analyzed as consequences than as exposures although they influence both ^17^.

Social factors are deemed important when working with holistic health frameworks and many see that this is an important area to invest effort across research themes. Though globally few of the research papers analyzed have carried comprehensive analyses of the diverse inter-relations with climate change and health reflecting a still largely incomplete knowledge. Considering the role of social factors in structuring the environmental exposition, people resilience and ability to cope, it is crucial they are considered in a more systematic way and further studied as the main outcome and variable of interest to understand the underlying mechanisms of construction. Only such an approach to the social environment will provide some factual clarification and guide how to possibly reduce the aggravating effects or prevent new social inequalities. A set of methodological recommendations provided by Neufcourt *et al.* ^18^ can help extend research into this direction.

### Visualizing the challenging entanglement of social components in climate & health

**Figure 1.**
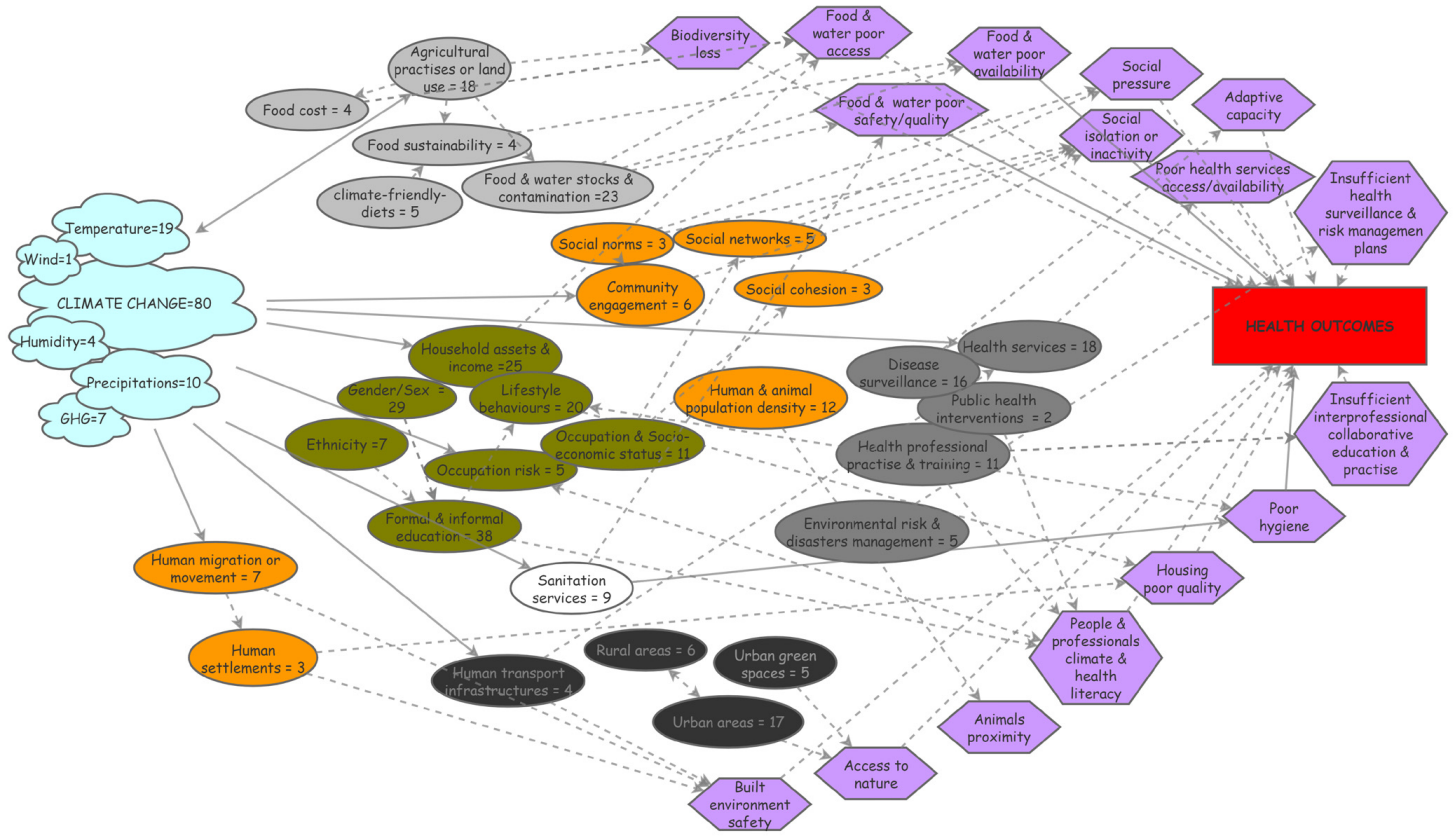
Process diagram figuring climate exposures, social components and their pathways to health. This diagram relies on 80 empirical papers identified in a literature review and individually scrutinized for text elements related to climate change or to social components that were either studied or mentioned; identified elements were reported in the diagram as cloud for climate elements or oval boxes for social ones (food-related in grey; health services-related in dark grey; individual characteristics in green; …). Theoretical pathways the most likely to link the social components to health outcomes were reported in the diagram as purple hexagon boxes; arrows were placed between the related social components and their possible pathways to health (plain arrows) as well as between possibly inter-linked social components (dashed arrows). GHG: greenhouse gas.

As the social environment is considered to have a potential influence on health in many unclear ways, we endeavored to map the social environment & pathways discussed so far. Process diagrams are visual representations of the way in which interacting factors behave within a complex system; they are useful for summarizing and organizing information from interdisciplinary research and help identify data gaps or links ^19^. First, we identified the specific nature of climate and social/societal components discussed in the 80 empirical studies. It resulted in a number of contributive elements that we reported as a process diagram along with the corresponding number of studies they were part of ( Figure 1), to facilitate their apprehension. We then inferred the theoretical pathways most likely to relate the social components to health outcomes from the literature in social epidemiology and reported them in the diagram. Potential links were determined and placed between the related social components and their possible pathways as well as between some social components.

The climate elements were overall mainly referred to as the generic concept of ‘climate change’. Among the meteorological parameters studied, temperature was the most frequent component measured, followed by precipitations, greenhouse gas emissions and humidity; wind conditions were addressed in one study, which highlights the lack of research on some climate factors that could also impact health substantially ( Figure 1, cloud boxes).

Regarding social factors, six broad categories (visualized as colored clusters in the diagram, Figure 1) emerged from the analysis: factors related to food (light grey boxes) or health systems (dark grey boxes) and to individual characteristics (i.e., lifestyle behaviors, gender, education, livelihoods – green boxes) were most often reported, followed by factors related to built environment (black boxes) and to sanitation (white box). Their potential relations to health were analyzed and the most obvious theoretical pathways possibly mediating these effects were inferred from the social epidemiology literature. These pathways were integrated into the diagram as hexagons and their relationships as arrows (plain arrows for direct effects, dashed arrows for indirect effects; Figure 1). This knowledge thus synthesized can be visualized more easily and may be helpful to inform and guide research in identifying upstream risk factors and eventually providing a way to conceptualize causal processes.

### Framing and structuring the complexity

**Figure 2.**
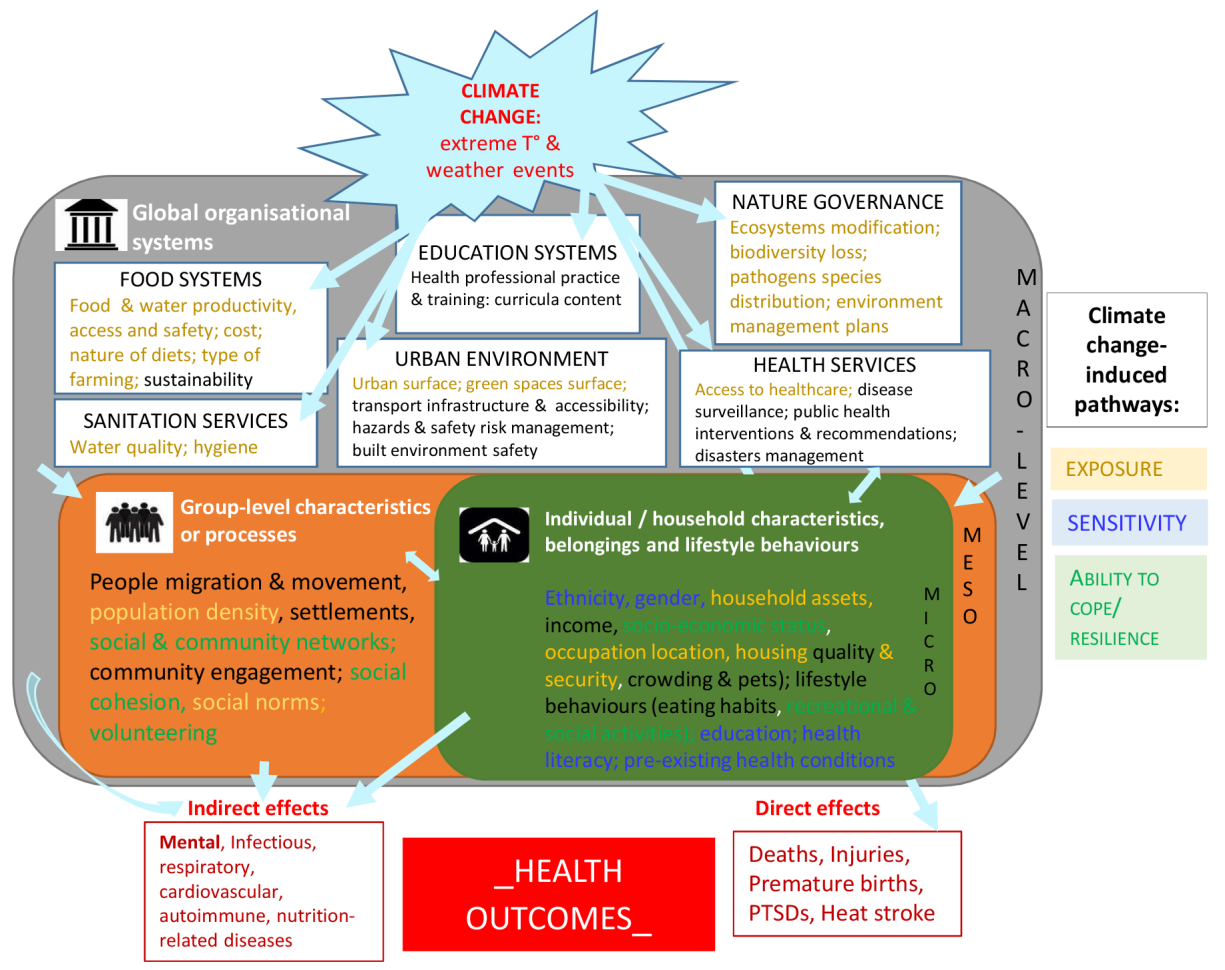
Synthetic knowledge framework for climate change, health outcomes and social determinants of health. This theoretical framework is based on the knowledge generated from the empirical studies describing the inter-dependence between climate change, health outcomes and social determinants of health at three different social organization levels (micro/meso/macro). T°: temperature; PTSD: post-traumatic syndrome disorder.

Such inter-relations between climate change, social conditions or organization, and health outcomes remain complex and are a key hurdle in addressing social inequalities. Clarifying the research-based knowledge by further structuring all the information identified as a knowledge framework may help facilitate the analysis and the understanding of underlying structural systems. Figure 2 provides such a framework where the diversity and heterogeneity of the identified social components were categorized and organized as various layers of social characteristics and/or social organization constitutive of distinct levels of the environment (micro/meso/macro-environments), all relating in some way to key pathways of climate change impacts. The micro-level consisted of all individual or household characteristics such as ethnicity, gender, household assets (defined as a broad category including livelihoods, social & socio-economic status, housing & pets, belongings and lifestyle behaviors like eating habits); the meso-level included characteristics or processes at a larger group-level such as people migration & movement, population density, settlements, social & community networks and engagement, professional practice & training for specific groups; the macro-level referred to global organizational systems or activities built by the society such as food systems, sanitation & health services, education, built environment, risks management. Such a framework can be used by researchers and health actors interested in understanding social determinants and consequences of climate change on health, and facilitate a more systematic consideration and integration of social variables in our understanding, mitigation or prevention of the mechanisms underlying the relationship between climate change and health.

### Data challenges

Another key hurdle explaining the difficulty in addressing social factors is the lack of data and lack of data linkage. Social data are either rarely or not measured, or are not reusable or linkable across health, social and climate disciplines. The reuse from multi-sources databases and linkage of individual-level data remains challenging at several levels: difficult discoverability; data heterogeneity between sources; lack of social data collection; frequent impossible linkage between databases; complex legal processes for data access and reuse; unwillingness from some researchers to share data; and considerable work needed for data preparation before sharing, not anticipated nor funded. These challenges, detailed in Box 1, are in our view major issues to be anticipated for further improvement ^20^.

**Box 1. Data challenges for reusing multi-sources databases and linkage of individual-level data**

i/ **Difficult discoverability:** Health & social data are quite fragmented and distributed across several information systems (healthcare establishments, research laboratories, public statistical databases, or many different governmental establishments) and in disciplinary siloes which makes it difficult to discover and access them. Climate model data have though been made openly available as part of the World Climate Research Program to researchers from a wide range of communities ^22^. There is a need for better data visibility;

ii/ **Increasing heterogeneity** of data from different sources: the information collected is increasingly heterogeneous due to (a) the varied structural and lexical nature of data (i.e. genomics, physiological, biological, clinical, pharmacy, imaging, medico-economic, epidemiological data for health; psychological, social, cultural, geographical for social data ; space or surface borne-instrumental, paleoclimatic, satellite, and model-based sources for climate); (b) the heterogenous formats of data which are not always easy to use for research purposes ( text, numerical values, signals, 2D and 3D images, genomic sequences, etc.); (c) the heterogenous data quality and their various levels of sensitivity;

iii/ **Data collection gap**: there is a lack of social data collection, e.g. social data characterizing people's living conditions & ethically collected data on race/ ethnicity which often do not exist in many countries for historical and cultural reasons, regarding e.g. racial discrimination, thus impeding the monitoring of health inequalities; on the other hand, climate data are well collected but increasing volumes of climate modelling are produced that complexifies their use ^22^;

iv/ **Inability to link databases**: Quality quantitative social data available at the individual level (i.e. an individual’s occupation, education level, income) are often not linked or linkable to quality health data across countries;

v/ **Complex legal processes** for data access and reuse: Due to the sensitivity of health & social data and to legal aspects such as the European General Data Protection Regulation, linking information across multiple data sources requires approvals that are subject to long delays which in turn are difficult to align with project schedules and funding timelines;

vi/ **Data hogging**: Heterogeneous and unclear data management results in a lack of open sharing practices by default, which often happens in the absence of open data mandates from the funder or publisher;

vii/ **Burden of data preparation**: The heavy burden of data preparation needed for open access and reuse and the lack of support for those tasks discourage researchers to carry them out.

### How to move forward: global recommendations

New research is crucial in deciphering the pathways involved in order to provide the required knowledge for decision and action either preventive or adaptive. We encourage health researchers and practitioners to adopt a holistic view integrating the social environment when designing their projects and get all relevant partners and stakeholders across disciplines and sectors on board from conception. Our proposed thinking tools may help guide such trans-sectorial research and can also help in planning data collection and analysis, prevention, public health programming, vulnerability and risk assessment. Notably they could be used along with policy-making frameworks such as the Driving Force-Pressure-State-Exposure-Effect-Action (DPSSEA) framework widely used in European and international health assessments.

In an attempt to move those issues forward, we provide in Box 2 a number of recommendations towards various stakeholders in climate, environment and health research. They are meant to help conceptualize and design research as systems where the social environment is assessed and considered as part of any research project or intervention addressing climate change & health. Furthermore, we recommend that national research bodies design plans and set programs to develop cross-disciplinary integrated platforms for secure data access and analysis that provide data spaces where both health data and social data can be accessed, linked, used and analyzed safely by researchers. Some countries have started to develop infrastructures to facilitate the matching of health databases with other sources like cohorts and administrative data such as at the regional level in the Western Cape, South Africa ^21^; others start providing complex computer and statistical programs and algorithms to analyze large volumes of information [E.g., https://www.health-data-hub.fr/]. Such initiatives must be supported and developed in a sustainable way.

**Box 2. Recommendations to stakeholders in climate & health research to better consider social data into research & intervention**

**➣    To Researchers :**

▪      Leverage holistic frameworks (such as One Health, Planetary Health or EcoHealth) where social determinants are a key feature, to guide the projects design and studies examining health in the context of climate change

▪      Use a systematic methodology to investigate at best social determinants potentially in relation with health and climate, as an environmental risk. A set of recommendations has been provided by Neufcourt *et al.* ^18^

▪      Use linked datasets across social, administrative, environmental and health sources where possible, thereby limiting selection bias, attrition and loss to follow-up which are difficult to manage in ad-hoc studies and adversely affect the inclusion of disadvantaged or vulnerable populations in analyses over time

**➣    To Health practitioners:**

▪      Use holistic frameworks such as those described in this paper as part of a global system thinking analysis to help design adapted interventions

▪      Systematically consider the living conditions of the target populations as part of a social risk assessment ahead of designing interventions

**➣    To Research institutions and funding agencies:**

▪      Actively promote sustainable health equity as a fundamental ethical principle that guides research policies and funding ^23^

▪      Decrease funding projects that do not prioritize sustainable health equity

▪      Call for more and strengthen the funding of global multidisciplinary holistic by design projects, in order to facilitate the connection of fields otherwise siloed

▪      Provide secure spaces to centralise multi-sources & multi-disciplinary data relevant for social epidemiology at relevant levels (local, national, European, regional…)

▪      Support a better visibility of health & social data: more catalog-like and integrated platforms

▪      Simplify health & social data access processes (rules & practicalities)

▪      Make all climate model data, observations, and the software used for processing open, free and easily available to all users, through international agreements

**➣    To publishers, funders, Research institutions:** Promote & facilitate open data

▪      Provide dedicated human and material support, i.e. steward / managers to help with data access and pre-processing; tools to assist access & use

▪      Provide training for students and all staff for data vocabulary and format standards/ storage/ legal issues, data science

▪      Provide recognition mechanisms to foster open science in general (ongoing work, Research Data Alliance - SHARC interest group)

▪      Require easy access to all data associated with the papers they publish or the work they fund

▪      Guide authors towards trustworthy repositories where to deposit data

▪      Guide authors with regards to how to make their data FAIR (Findable, Accessible Interoperable, Reusable) ^24^

### Limitations

This work was intentionally limited to three main holistic approaches of health because they offer the broadest framework to study environmental changes, including social ones, and as such, provided an overview of research committed to address climate change and the social determinants of health. Nevertheless, we are much aware that a substantial body of research has been carried out on the effects of climate change on health without explicitly referring to holistic approaches, including work on extreme heat in various contexts done by projects such as CHAMNHA, HEATCOST, CLIM-App, EXHAUSTION, and HEAT-SHIELD, and such papers were not included in our review.

The key large international reports from IPCC (2022) and WHO (2008, 2023) which were not present in the source databases (PubMed and Web of Sciences) were not included in the quantitative analysis, although they may have contained useful articles. Nevertheless, we included these sources in the discussion of results. Finally, we did not consider works that were not published in either English or French due to language constraints in the review team. Only a few such papers were identified, although they might be very relevant.

## Conclusions

In conclusion, holistic approaches to health such as One Health, EcoHealth or Planetary Health are frameworks well suited to investigate climate change and social determinants of health. However, our literature analysis highlighted that there was limited research using or referring to these approaches to study social determinants as the main variables of interest; individual social determinants were mostly absent or underrepresented in these studies. Hence, subsequent decision making and actions cannot adequately consider the social effects that drive climate change impacts on health and exacerbate climate change itself, leading too often to unsustainable or unfair solutions that may maintain the status quo.

We believe the reasons for these gaps are twofold: first, climate change impacts on health outcomes in a very complex manner, where inter-dependent relations can lead to negative uncontrolled feedback loops. This complexity makes it difficult to design comprehensive studies. Thinking tools can help organize concepts, ideas and relationships to better integrate and comprehensively understand the inter-dependencies at play thereby enabling adequate analysis for appropriate actions. We provide such tools here including a pathway diagram and a knowledge framework where social determinants are embedded in climate change impacts at three different actionable levels (micro, meso and macro levels), themselves driven by specific processes or resulting from different governance systems. Second, social data are difficult to measure, find or obtain, while health data are difficult to share and climate model data harder to reuse. Global effort is urgently needed to collectively organize and solve these hurdles. Transformative solutions to halt the effects of climate dysregulation and preserve the health of our planet and of its inhabitants will fail unless social inequalities are addressed as an integral part of the climate change reversal roadmap ^4^. Our findings are by no means exhaustive, but offer an overview of these important challenges for climate science and public health.

## Data Availability

All data underlying the results are available as part of the article and no additional source data are required.
