# Supplementary material for: On the need to better integrate the social environment in research on climate change and health: recommendations and thinking tools
Source: Open Res Eur. 2025 Jan 14;4:105. Originally published 2024 May 28. [Version 4] doi: 10.12688/openreseurope.17528.4 (PMC11736555; doi:10.12688/openreseurope.17528.4)
Supplement: Peer review history

# Reviewer report: Reviewer response for version 4

**DOI:** 10.21956/openreseurope.20941.r50460

**Published:** 2025-02-19

**Authors:** Rhiannon Thompson

**Affiliations:** 1 Imperial College London, London, UK

**General comments :**

 Overall, the manuscript makes some very intelligent points and is well justified in the current context, but sometimes the complexity of language, the choice of structure and the design of the figures make it hard work to follow/understand, which I think hinders accessibility, engagement, and reach.

 Structurally, to me it would make more sense for what’s found at the start of the discussion, where the approach is outlined, to go into a method section, followed by a results section with the percentages and figures, and then for the discussion section to include the content found from ‘data challenges’ onward.

**Specific comments:**

Introduction

 The title says 'social environment', but you also talk about social inequalities, social injustice, social conditions, social determinants of health (which is what's talked about in the conclusions section)... I note that in the discussion section you mention “ *The broadly-defined social environment - i.e. related to any social organization, process, norm, habits, and characteristics possibly affecting health*" but this still feels quite vague and I'm not sure of the parameters of what you're talking about most of the time. To guide the reader, the introduction would benefit from a clear definition of the scope of this review, with examples.

Discussion

 I would consider checking the grammar in this sentence: “ *Considering the role of social factors in structuring the environmental exposition, people resilience and ability to cope*”.

 Figure 1: I appreciate the complexity of these relationships is reflected in this very complex diagram but I find this diagram difficult to take in and interpret. I think it should be larger, maybe presented as a whole landscape page, and the dark grey (health services) green (individual characteristics) and black (?) boxes are hard to read- can you select more high contrast shades/colour combinations here? It would also help if all colours/shapes were defined in the legend, and some of the arrows are simply too clumped together to easily delineate which are which.

 Figure 2: I also find this figure confusing, I can't really tell which arrows are connecting which sections. Is the top climate change, the middle social determinants and the bottom health outcomes? I think this could also be better designed and labelled.

 I like the recommendations box, it’s good to have clear identifiable and actionable steps arising from the analysis for different stakeholders.

Is the review written in accessible language?

Partly

Are all factual statements correct and adequately supported by citations?

Yes

Are the conclusions drawn appropriate in the context of the current research literature?

Yes

Is the topic of the review discussed comprehensively in the context of the current literature?

Yes

Reviewer Expertise:

Public health, epidemiology, review methods, systematic reviews, mixed methods, mental health, climate change, physical environment

I confirm that I have read this submission and believe that I have an appropriate level of expertise to confirm that it is of an acceptable scientific standard, however I have significant reservations, as outlined above.

# Reviewer report: Reviewer response for version 4

**DOI:** 10.21956/openreseurope.20941.r49784

**Published:** 2025-02-05

**Authors:** Panagiota Kotsila

**Affiliations:** 1 Universitat Autònoma de Barcelona, Barcelona, Spain

The article correctly points to a gap in the literature, whereby the extent to which research approaches based on holistic frameworks around health (OneHealth, Planetary Health etc.) investigate social determinants of health, is not clear. It seeks to review the ways in which the social environment is used to better understand the relationship between climate and health. 

 If OneHealth and EcoHealth are not frameworks that invite much discussion on the socio-political and cultural systems that define health outcomes, why not focus the article on how Planetary Health can better integrate a social determinants of health analysis?

 In general, the "social environment" is not a concept that is explained much in this article and it needs to be acknowledged that "any social organization, process, norm, habits, and characteristics possibly affecting health (directly or indirectly) including lifestyle behaviors" is a very narrow definition, and if those terms were used in the search, they are excluding, for example, things such as "power imbalance", "inequality", "justice".  There is plenty of literature in the critical environmental humanities on this, and it should be acknowledged. While there might not be space, and not the aim of this paper to integrate this literature here, it should be acknowledged that environmental and climate justice scholarship has long been investigating these questions in relation to health.  

 The graph could be improved to communicate these complex relationships and be visually clearer. A lot of these elements (e.g. sanitation) or theoretical frameworks, as the authors call them, (e.g. food and water poor availability), have many layers, express in various scales differently, and have further drivers or determinants that depend on socio-economic and cultural systems and which are not engaged with or discussed. Literature for example on political ecologies of risk can help unpack this further.  Since you are using literature beyond the articles in the review to construct this graph, you might as well use some of the more critical humanities theoretical contributions, and signal this in the graph (which aspects come from lit review and which are from your own reading of the literature beyond the search). 

 When referring to data challenges, the focus is on quantitative data, but qualitative social research can be of great use in documenting and explaining some of these relationships. 

 "These are meant to be used when designing empirical studies aimed at investigating relations between exposome and health outcomes" --> check grammar.

Is the review written in accessible language?

Yes

Are all factual statements correct and adequately supported by citations?

Partly

Are the conclusions drawn appropriate in the context of the current research literature?

Partly

Is the topic of the review discussed comprehensively in the context of the current literature?

Partly

Reviewer Expertise:

political ecology; climate justice; urban greening; critical public health

I confirm that I have read this submission and believe that I have an appropriate level of expertise to confirm that it is of an acceptable scientific standard, however I have significant reservations, as outlined above.

# Reviewer report: Reviewer response for version 3

**DOI:** 10.21956/openreseurope.20514.r46928

**Published:** 2025-01-16

**Authors:** Sandrah P Eckel

**Affiliations:** 1 University of Southern California, Los Angeles, California, USA

Thank you to the authors for your responses to my comments, I have no further suggestions.

Is the review written in accessible language?

Yes

Are all factual statements correct and adequately supported by citations?

Yes

Are the conclusions drawn appropriate in the context of the current research literature?

Yes

Is the topic of the review discussed comprehensively in the context of the current literature?

Yes

Reviewer Expertise:

Biostatistics, Environmental Health, Climate and Health

I confirm that I have read this submission and believe that I have an appropriate level of expertise to confirm that it is of an acceptable scientific standard.

# Reviewer report: Reviewer response for version 3

**DOI:** 10.21956/openreseurope.20514.r46927

**Published:** 2025-01-07

**Authors:** Rosemary Hiscock

**Affiliations:** 1 University of Bath, Bath, England, UK

I have no further comments.

Is the review written in accessible language?

Yes

Are all factual statements correct and adequately supported by citations?

Partly

Are the conclusions drawn appropriate in the context of the current research literature?

Partly

Is the topic of the review discussed comprehensively in the context of the current literature?

Partly

Reviewer Expertise:

NA

I confirm that I have read this submission and believe that I have an appropriate level of expertise to confirm that it is of an acceptable scientific standard.

# Reviewer report: Reviewer response for version 3

**DOI:** 10.21956/openreseurope.20514.r47466

**Published:** 2024-12-24

**Authors:** Roger Few

**Affiliations:** 1 School of International Development, University of East Anglia, Norwich, England, UK

This paper has been reviewed previously by two peer reviewers, and a report on the responses to their comments provided. I have reviewed both the paper itself and I am satisfied that the author has responded positively and sufficiently to the comments. In light of this, I am happy to endorse the paper for publication as a positive contribution to the field, helping to shed light on the complexities of considering social dimensions within ecologically-framed analyses of the linkages between climate change and health. 

 I have no substantial further comments, other than two things I would like to see addressed in the Introduction (which is key because it sets the orientation of the piece):

 1) Define/introduce the term 'dysregulation' more informatively, and also any critiques of the term. To me, it is not a well-known term as used in the context of climate change.

 2) Please be careful in the phrasing of this sentence in the second paragraph, starting 'Climate change has adversely impacted....'.  because I feel the wording gives the impression that climate change is solely responsible for these environmental risk-related impacts on health. Of course, it is not - e.g. as noted in the opening sentence, extreme weather events will exist regardless of climate change's exacerbating effect on their frequency, magnitude or distribution. The authors know this, but referring again to the contributory or amplifying effect of CC in this sentence would be wise, I feel.

Is the review written in accessible language?

Yes

Are all factual statements correct and adequately supported by citations?

Yes

Are the conclusions drawn appropriate in the context of the current research literature?

Yes

Is the topic of the review discussed comprehensively in the context of the current literature?

Yes

Reviewer Expertise:

Climate change, disaster risk, health, vulnerability and adaptation

I confirm that I have read this submission and believe that I have an appropriate level of expertise to confirm that it is of an acceptable scientific standard.

# Response

**Published:** 2025-01-08

**Authors:** Laurence Mabile

**Affiliations:** CERPOP, Universite Toulouse III Paul Sabatier, Toulouse, Occitanie, France

Many thanks for your encouraging remarks and relevant suggestions that help us to be more accurate. 

 From Reviewer 3:

 I have no substantial further comments, other than two things I would like to see addressed in the Introduction (which is key because it sets the orientation of the piece):

 1) Define/introduce the term 'dysregulation' more informatively, and also any critiques of the term. To me, it is not a well-known term as used in the context of climate change.

**Author Response:** Climate dysregulations are meant as climate changes induced by human activity that per se disturb the natural phenomena and amplify extreme responses. This is now specified in the corresponding sentence (Introduction, paragraph 1, line 4).

 2) Please be careful in the phrasing of this sentence in the second paragraph, starting 'Climate change has adversely impacted....'.  because I feel the wording gives the impression that climate change is solely responsible for these environmental risk-related impacts on health. Of course, it is not - e.g. as noted in the opening sentence, extreme weather events will exist regardless of climate change's exacerbating effect on their frequency, magnitude or distribution. The authors know this, but referring again to the contributory or amplifying effect of CC in this sentence would be wise, I feel.

**Author Response:** The phrasing has been modified as follows: "Climate change has contributed to adversely impact health in many ways..." (Introduction, paragraph 2, line 3).

# Reviewer report: Reviewer response for version 1

**DOI:** 10.21956/openreseurope.18943.r43192

**Published:** 2024-08-29

**Authors:** Rosemary Hiscock

**Affiliations:** 1 University of Bath, Bath, England, UK

This paper looks at how previous research using holistic models has linked worsening socioeconomic inequalities in health to climate change.

 The article is a big overview but at times is too non specific

 Box2 the list of problems with getting data was useful.

 The paper needs to talk more about holistic theories and introduce systems theory – concepts from which explain why holistic theories are important.  The paper could make more of negative feedback loop of climate change leading to more inequality which then leads to more climate change

 The paper needs to conceptualies socioeconomic position more precisely.  Socioeconomic position is income and wealth, occupation, education.  Ethnicity and gender are not socioeconomic position.  They are demographic characteristics which can predispose towards lower socioeconomic position.  Then there are conditions associated with low socioeconomic position eg poor condition insecure housing, fewer consumer goods including cars.  SOMETIMES low socioeconomic position is associated with poor access to healthy food, transport, internet, education however in other cases poor people live in central cities with good access. 

  People who can’t afford to eat won’t be able to afford more climate friendly options or alternatively are more environmentally friendly because they can’t afford as much meat or air-freighted vegetables flown in from great distance.  Then there are cultural attitudes where low socioeconomic position might not see being climate aware as for them or they can’t socialise with their peers if they refuse to eat fast food McDonalds etc – peer support may be more important for low socioeconomic position in order to get by.

 In figure 1 and 2 there appear to be wider social determinants than just socioeconomic or sociodemographic inequality eg pets

 Also  inequalities between high income and low and middle income countries globally could be emphasised for a global approach. 

 There also appeared to be confusion between climate change, climate change adaption, climate change  mitigation and collateral damage from climate change policies

 I think the paper could do with a box with examples of empirical studies which showed

1. Climate change leads to worse inequality
2. Climate change mitigation/adapaption policies lead to worse inequality
3. Inequality leads to worse climate change

For each of these the main concepts of climate change inequality and linking concepts (eg health professional expertise) should be detailed

 Intro para 3 “Social position also affects”  might be better as “social position may be associated with poor health outcomes because it is linked to”

 Intro para 4

 “mudslides or close to waste sites, roads and factories” why is this linked to climate change?

 “typical daily tasks imposed by social norms, or some workers whose occupational location and activities, or populations living in poor quality housing or with comorbidities; and a decreased ability to cope with stressful events or to prevent or recover from physical and material damages (due to systematic resource-constraints such as lack of private resources, of insurance contracts, of access to public resources and services…)”  Again these are not themselves linked to climate change.

 “susceptibility of some population groups such as women” you are conflating socioeconomics with demographics here.  You need to say some demographic groups are overrepresented in proportion in low socioeconomic position such as women and some ethnicities in the previous paragraph to set this up.

 “These driving forces can lead to property loss, reduced livelihoods, residential relocation, and other climate-related crises (infrastructure damages limiting access to healthcare, food insecurity, forced migration, war…)”  I think you need a section on what climate related crises are before you link to low social position

 “some economic contexts” what contexts – are they common, are they likely to persist?

 “ As poverty and social injustice”  I think this is a new paragraph which needs to be started with “Climate change is likely to exacerbate inequality”

 “falling” change to “expected to fall”.  Define extreme poverty

 “Therefore, it is critical to understand the global picture when to design adequate actions, not to imbalance the system or generate deleterious effects in other places.”

 This conclusion does not follow from what you have written.  You would need to add info about poverty being in many different places globally and a bit about systems theory in order to understand why an action in one place might affect somewhere else and why they might not also be positive.

 Intro para 5 “As well as their own vulnerability to the effects of climate change, the most socially disadvantaged are also disproportionately burdened by new and often regressive policies which are set in place to foster behavioral or cultural changes” you need to reword to say that some climate change policies to foster behavioral or cultural changes can have regressive impacts

 “As such, understanding and identifying the social patterning of the human health and wellbeing consequences of climate dysregulation and ecosystem disruption are key if we are to avail ourselves of actionable, reliable research and policy priorities ^14^. “  This sentence does not follow from the previous sentence– this sentence is about the impact of climate change whereas the previous sentences were about impacts of policies to combat climate change.

 Intro 1 para 6 Can you say what the differences between the 3 approaches are

**Climate change, social environment & health within holistic frameworks: insights from the literature analysis**

 Para 1 ‘infiltrating’ sounds like an enemy invading.

 Might be good to include here if you searched databases eg pubmed

 I would say this paragraph is a methods not a discussion section – the following paragraphs are results not a discussion

 Figure 1 gender and ethnicity are not themselves socioeconomic status – they are demographic behavours where some subgroups are overrepresented in low socioeconomic position. 

 Figure 2 appears to have lost sight of the concept of socioeconomic status.  It appears to confuse being low socioeconomic status and behaviours that go with it (eating habits) and living conditions (housing) and values (education)

 Limitations

 You need to set up better in the introduction what holistic approaches are and why you think they are most useful.

 You also need to include in the limitations that you have not included commercial determinants of health which impact climate change and social inequalities  and government power to reduce inequalities and climate change  eg https://gh.bmj.com/content/8/11/e013698 https://gh.bmj.com/content/8/11/e013698.abstract

 https://blogs.bath.ac.uk/tcrg/2023/04/06/why-cdoh-matters/

Is the review written in accessible language?

Yes

Are all factual statements correct and adequately supported by citations?

Partly

Are the conclusions drawn appropriate in the context of the current research literature?

Partly

Is the topic of the review discussed comprehensively in the context of the current literature?

Partly

Reviewer Expertise:

health inequalities, commercial determinants of health, climate change, tobacco supply chain

I confirm that I have read this submission and believe that I have an appropriate level of expertise to state that I do not consider it to be of an acceptable scientific standard, for reasons outlined above.

# Response

**Published:** 2024-11-14

**Authors:** Laurence Mabile

**Affiliations:** CERPOP, Universite Toulouse III Paul Sabatier, Toulouse, Occitanie, France

Authors point-by-point responses to Reviewer 2:

 First, many thanks for your scrutiny and all your valuable comments. Please find our point by point responses below. Accordingly, we have integrated in the revised version 2 as many relevant changes as possible given the wordcount constraint.  

Reviewer comments: This paper looks at how previous research using holistic models has linked worsening socioeconomic inequalities in health to climate change. The article is a big overview but at times is too non specific

Authors Response: As this first statement has generated a number of comments, we would like to clarify from the start that our work did not focus specifically on socio-economic inequalities but more broadly on the social environment that affects health, as stated in the title of our manuscript and specified in the first paragraph of our Discussion section, i.e. “Among articles which simultaneously included i) the term “climate change”, ii) one of the holistic above-mentioned frameworks (OH, EcoH or PH) and iii) at least one term related to the broadly-defined social environment - i.e. related to any social organization, process, norm, habits, and characteristics possibly affecting health (directly or indirectly) including lifestyle behaviors -, we first found… ».  Accordingly the keywords string we used for the bibliographic database search included socio-economic components such as income, occupation and education but also age, gender, ethnicity, living conditions, nutrition and others at the macro level. The complete string is available at [https://osf.io/bjsua]. We have now clarified this information in the Data availability section.

Reviewer comments: Box2 the list of problems with getting data was useful.

Authors Response: Thank you for your encouragements.

Reviewer comments: The paper needs to talk more about holistic theories and introduce systems theory – concepts from which explain why holistic theories are important. 

Authors Response: The following sentences have been added in Introduction, ahead of the description of holistic models (paragraph 6):   “Given the complexity of the climate and health challenges that span various knowledge disciplines, necessitating multi-level analyses and the involvement of diverse sectoral actors, the importance of systems thinking has emerged. Unlike traditional statistical analysis, which tends to isolate individual components of study, systems thinking views these components as a complex whole made up of interrelated and interdependent parts (ref  URLs: https://dx.doi.org/10.4135/9781529730739; https://doi.org/10.1093/heapol/czs088; https://doi.org/10.1007/978-3-319-00104-3_3). This shift has led to the rise of holistic health approaches over the past decade, including EcoHealth, One Health, and more recently, Planetary Health…”  

Reviewer comments: The paper could make more of negative feedback loop of climate change leading to more inequality which then leads to more climate change.

Authors Response: We tackled those points in Introduction. However, results from the scoping review did not bring about such feedback elements driving us to discuss other aspects.

Reviewer comments: The paper needs to conceptualies socioeconomic position more precisely.  Socioeconomic position is income and wealth, occupation, education.  Ethnicity and gender are not socioeconomic position.  They are demographic characteristics which can predispose towards lower socioeconomic position.  Then there are conditions associated with low socioeconomic position eg poor condition insecure housing, fewer consumer goods including cars.  SOMETIMES low socioeconomic position is associated with poor access to healthy food, transport, internet, education however in other cases poor people live in central cities with good access. 

  People who can’t afford to eat won’t be able to afford more climate friendly options or alternatively are more environmentally friendly because they can’t afford as much meat or air-freighted vegetables flown in from great distance.  Then there are cultural attitudes where low socioeconomic position might not see being climate aware as for them or they can’t socialize with their peers if they refuse to eat fast food McDonalds etc. – peer support may be more important for low socioeconomic position in order to get by.

 In figure 1 and 2 there appear to be wider social determinants than just socioeconomic or sociodemographic inequality eg pets

Authors Response: Although the perspective of our work was not to focus specifically on socio-economic factors, we agree that some parts of the text might have been confusing. This has been clarified in paragraph 3 by adding the following text: “as for socio-economic factors, the poorest people have the highest levels of morbidity and premature mortality” “Social position includes social components such as age, gender and ethnicity, and thereby thereby may be associated with poor health outcomes because of its relationship with other determinants of health,…” The WHO conceptual Framework for Action on the Social Determinants of Health has been added as a reference at the end of the paragraph: Solar et al. 2010 - https://iris.who.int/bitstream/handle/10665/44489/9789241500852_eng.pdf?sequence=1

Reviewer comments: Also inequalities between high income and low and middle income countries globally could be emphasized for a global approach. 

Authors Response: The following sentence has been added to the section related to systems thinking, paragraph 7 of introduction: “Specifically, these frameworks are well suited to tackle inequalities between high and low and middle income countries”

Reviewer comments: There also appeared to be confusion between climate change, climate change adaption, climate change  mitigation and collateral damage from climate change policies

Authors Response: In our work, the term climate change referred to meteorological factors and sometimes to related extreme events. When referring to adaptation or mitigation, this was specified.

Reviewer comments: I think the paper could do with a box with examples of empirical studies which showed

1. Climate change leads to worse inequality
2. Climate change mitigation/adaptation policies lead to worse inequality
3. Inequality leads to worse climate change

For each of these the main concepts of climate change inequality and linking concepts (eg health professional expertise) should be detailed

Authors Response: As highlighted by our work, social factors are investigated empirically only in a minor fraction of studies addressing the relation between climate and health in holistic frameworks. None of the factors related to individual characteristics (gender, ethnicity, education) or to household assets (including occupation tenure and socio-economic items) were addressed as an exposure or outcome which makes it impossible to provide the requested table. Some examples of empirical studies are detailed in supplementary material (https://osf.io/6t3j7). However, we agree that your three suggested points would gain from being more emphasized in introduction. Accordingly, we have added the following references (& text) in the indicated sections of Introduction :

1. Climate change leads to worse inequality:

Text added in paragraph 4 : « Additionally, climate changes can worsen income or occupation inequality and may even create a feedback loop with it « (Malam et al. 2017: https://doi.org/10.1504/IJESD.2017.080848; Ogbeide-Osaretin et al; 2022: https://doi.org/10.32479/ijeep.13556; Fitzgerald et al. 2022: https://doi.org/10.1016/j.erss.2021.102385) »

 2.Climate change mitigation/adaptation policies lead to worse inequality: References added in paragraph 6 : Niiwul et al. 2021: https://doi.org/10.1016/j.jclepro.2020.123393;   Bae et al. 2018: http://keapaper.kea.ne.kr/RePEc/kea/keappr/KER-20180701-34-2-04.pdf

 3. Inequality leads to worse climate change: References added in paragraph 4: Ogbeide-Osaretin et al; 2022: https://doi.org/10.32479/ijeep.13556; Yameogo et al. (2020), The effect of income inequality and  economic  growth  on  environmental  quality:  A  comparative  analysis between Burkina Faso and Nigeria. Journal of Public Affairs, 7(2), 148-163.  

Reviewer comments: Intro para 3 “Social position also affects” might be better as “social position may be associated with poor health outcomes because it is linked to”

Authors Response: Changes have been made in the text, the sentence is now as follows: “Social position includes social components such as age, gender and ethnicity,  and thereby may be associated with poor health outcomes because of its relationship with other determinants of health, such as…”

Reviewer comments: Intro para 4:

 “mudslides or close to waste sites, roads and factories” why is this linked to climate change? Authors Response: These are indirect consequences of climate change. Mudslides are more likely to occur following flooding events which are part of the extreme events triggered by climate change; Proximity to waste sites increases risk of contamination in case of flooding for instance.

Reviewer comments:  “typical daily tasks imposed by social norms, or some workers whose occupational location and activities, or populations living in poor quality housing or with comorbidities; and a decreased ability to cope with stressful events or to prevent or recover from physical and material damages (due to systematic resource-constraints such as lack of private resources, of insurance contracts, of access to public resources and services…)”  Again these are not themselves linked to climate change.

Authors Response: Climate extreme events are more stressful and impactful to poor populations who have been shown to be less resilient, i.e. have a decreased ability to cope with, prevent or recover from such events. In other words, as external stressors, climate events mean disadvantaged populations are exposed to physiological stress responses, and degraded material conditions, both of which are mechanisms driving health inequalities. The word climatic has been added as follows: “decreased ability to cope with stressful climatic events 

Reviewer comments:  “susceptibility of some population groups such as women” you are conflating socioeconomics with demographics here.  You need to say some demographic groups are overrepresented in proportion in low socioeconomic position such as women and some ethnicities in the previous paragraph to set this up.

Authors Response: Gender & ethnicity may be related to socio-economic status, however they are structural systems that are connected to and interact with socio-economic factors. They systematically assign roles in society such as for example outdoors daily tasks for African women or road works for male immigrants.

Reviewer comments:  “These driving forces can lead to property loss, reduced livelihoods, residential relocation, and other climate-related crises (infrastructure damages limiting access to healthcare, food insecurity, forced migration, war…)”  I think you need a section on what climate related crises are before you link to low social position

Authors Response: We have provided additional references to climate related crises in the following sentence: “These driving forces can lead to property loss, reduced livelihoods, residential relocation, and other climate-related crises (infrastructure damages limiting access to healthcare, food insecurity, forced migration, war…) further widening inequalities (URLs: Markova et al., 2018 - http://www.fao.org/3/I8656EN/i8656en.pdf ; Vuong et al. 2024 - https://doi.org/10.1007/s00267-024-01976-4; Ribot et al, 2023 - https://doi.org/10.1093/oxfordhb/9780197618646.013.20, accessed 26 Oct. 2024)”.

Reviewer comments:  “some economic contexts” what contexts – are they common, are they likely to persist?

Authors Response: This has been clarified as follows: “On top of this, social inequalities and per capita greenhouse gas emissions are positively correlated in emerging economies…”

Reviewer comments:  “ As poverty and social injustice”  I think this is a new paragraph which needs to be started with “Climate change is likely to exacerbate inequality”

Authors Response: We have modified the paragraph accordingly.

Reviewer comments:  “falling” change to “expected to fall”.  Define extreme poverty

Authors Response: This has been changed and clarified in the text as follows: “According to the World Bank, the estimated number of people expected to fall into extreme poverty (i.e., income below the international Poverty line) due to climate change is ranged between 32 million and 132 million in most scenarios by 2030”.

Reviewer comments:  “Therefore, it is critical to understand the global picture when to design adequate actions, not to imbalance the system or generate deleterious effects in other places.”

 This conclusion does not follow from what you have written.  You would need to add info about poverty being in many different places globally and a bit about systems theory in order to understand why an action in one place might affect somewhere else and why they might not also be positive.

Authors Response: The sentence has been modified as follows for more clarification: “Therefore, it is critical to understand the global picture to design adequate actions and break the devastating loop.”

Reviewer comments: Intro para 5 “As well as their own vulnerability to the effects of climate change, the most socially disadvantaged are also disproportionately burdened by new and often regressive policies which are set in place to foster behavioral or cultural changes” you need to reword to say that some climate change policies to foster behavioral or cultural changes can have regressive impacts

Authors Response: Changes have been made in the text accordingly. The sentence is now: “As well as their own vulnerability to the effects of climate change, the most socially disadvantaged are also disproportionately burdened by new policies which are set in place to foster behavioral or cultural changes and which can have regressive impacts”. Following, two references have been added: Nyiwul 2021: https://doi.org/10.1016/j.jclepro.2020.123393 Bae et al. 2018: Impacts of income inequality on CO2 emission under different climate change mitigation policies. Korean Econ Rev, 34(2), 187-211 (2018).

Reviewer comments:  “As such, understanding and identifying the social patterning of the human health and wellbeing consequences of climate dysregulation and ecosystem disruption are key if we are to avail ourselves of actionable, reliable research and policy priorities ^14^ . “This sentence does not follow from the previous sentence– this sentence is about the impact of climate change whereas the previous sentences were about impacts of policies to combat climate change.

Authors Response: We have clarified the meaning as follows: “As such, understanding and identifying the social patterning of the human health and wellbeing consequences of climate dysregulation and ecosystem disruption and of ensuing incentives or deterrent measures are key if we are to avail ourselves of actionable, reliable research and appropriate policy priorities”.

Reviewer comments: Intro 1 para 6 Can you say what the differences between the 3 approaches are

Authors Response: The overall differences between the three holistic approaches lie in which environmental aspects they most emphasise when framing human health: interactions of human with all species within ecosystems as for EcoHealth; interactions between animals & human as for One Health; limited resources of the planet as for Planetary Health. These differences are already highlighted in paragraph 7 (previously 6).

Reviewer comments: Climate change, social environment & health within holistic frameworks: insights from the literature analysis Para 1 ‘infiltrating’ sounds like an enemy invading.

Authors Response:  “Infiltrating” has been changed by “infusing”.

Reviewer comments: Might be good to include here if you searched databases eg pubmed Authors Response: This is now specified in the text and detailed in supplementary data as mentioned.

Reviewer comments: I would say this paragraph is a methods not a discussion section – the following paragraphs are results not a discussion

Authors Response: We are constrained by the editorial structure of ORE reviews which do not include the usual Method and Results section. We have thus detailed the methodology as supplementary data on OSF and discussed results and beyond in the Discussion section.

Reviewer comments: Figure 1 gender and ethnicity are not themselves socioeconomic status – they are demographic behaviors where some subgroups are overrepresented in low socioeconomic position. 

Authors Response: We fully agree. As mentioned earlier, our perspective was to study social components in a broad sense (as defined in the text) that includes gender and ethnicity as they are structuring factors in building social patterns of health.

Reviewer comments: Figure 2 appears to have lost sight of the concept of socioeconomic status.  It appears to confuse being low socioeconomic status and behaviors that go with it (eating habits) and living conditions (housing) and values (education)

Authors Response: We have intentionally identified all the social elements (in the broad sense defined in our text) that resulted from the scoping review to provide a synthetic scheme helping view in one shot all involved elements. This scheme only attempts to organise these elements as meaningful levels (micro/meso/macro) of influence and interactions with climate change and health but not in a causal way. Socio-economic status is related to behaviours, living conditions and education (at the micro-level). This framework does not distinguish structuring factors of social inequalities of health such as socio-economic ones or education from more distal factors like living conditions or behaviors as described by Solar O, Irwin A.[ A conceptual framework for action on the social determinants of health. WHO Social Determinants of Health Discussion Paper 2 (Policy and Practice). 2010 ]

Reviewer comments: Limitations

 You need to set up better in the introduction what holistic approaches are and why you think they are most useful.

Authors Response: We have clarified this at the beginning of paragraph 7 while adding the section on systems thinking above mentioned. The text is now: “Given the complexity of the climate and health challenges that span various knowledge disciplines, necessitating multi-level analyses and the involvement of diverse sectoral actors, the importance of systems thinking has emerged. Unlike traditional statistical analysis, which tends to isolate individual components of study, systems thinking views these components as a complex whole made up of interrelated and interdependent parts (Yasobant et al. 2020; Atun, 2012; Koskinen, 2013 = new refs 27-29. This shift has led to the rise of holistic health approaches over the past decade, including EcoHealth, One Health, and more recently, Planetary Health…”

Reviewer comments: You also need to include in the limitations that you have not included commercial determinants of health which impact climate change and social inequalities  and government power to reduce inequalities and climate change  eg https://gh.bmj.com/content/8/11/e013698 https://gh.bmj.com/content/8/11/e013698.abstract

https://blogs.bath.ac.uk/tcrg/2023/04/06/why-cdoh-matters/

Authors Response: We fully agree that commercial determinants are key components of the climate change/social inequality/health landscape but we did consider them as economic factors rather than social ones. The following sentence was added in the Limitations paragraph. “Furthermore, we have not included commercial determinants of health in the bibliographic search string related to social components despite their important role in climate change and social inequalities.”

# Reviewer report: Reviewer response for version 1

**DOI:** 10.21956/openreseurope.18943.r42200

**Published:** 2024-08-27

**Authors:** Sandrah P Eckel

**Affiliations:** 1 University of Southern California, Los Angeles, California, USA

Thank you for the opportunity to provide comments on this review article which is a call to action on better integrating the social inequities and societal factors into climate change and health research & policy response. The authors conducted a review of the literature at the intersection of climate change, holistic frameworks of health and the social environment, summarizing trends (and identifying gaps?) with the process diagram in Figure 1. The authors then structure this information in the synthetic knowledge framework in Figure 2, and provide a summary of barriers to progress in terms of data-related challenges (Box 1) and recommendations for various stakeholders (Box 2). The authors are to be commended for tackling such a large and tricky problem of how to advance the study of climate and health by integrating the social environment. This review will help drive progress, though I do have several comments which may help strengthen the contribution.

**Major comments:**

- It is challenging to operationalize a holistic approach in empirical studies, as shown by only 20% of the reviewed articles being empirical. The general recommendations for researchers in Box 2 are helpful. But it might drive the field forward more quickly to highlight several empirical research case studies (e.g., drawn from the 20%), perhaps sharing strengths/limitations.
- The recommendations of Neufcourt et al are mentioned twice, but not described. Please add a more context here for the reader.
- Epidemiological studies are increasingly using causal thinking and directed acyclic graphs (DAGs), can the authors provide an example of how to operationalize Fig 1&2 in an epi study DAG?
- As described meso-level processes seem well-suited to ecological analysis of administrative data, might be worthwhile to describe this more?
- Fig 1 – I was surprised to not see wildfires or air pollution in the climate clouds.

**Minor comments:**

 Introduction, Paragraph 6, Sentence 1: consider citing a review that compares the 3 holistic frameworks of health mentioned, perhaps: Lerner H, Berg C, 2017 [Ref-1]

 Discussion paragraph 1. Reconsider use of the word “significant” since readers may think this refers to statistical significance of a hypothesis test

 Fig 1/text – English language - consider using “precipitation” rather than “precipitations”

 Fig 2 – note: with current color choice some words become illegible when printed in black and white.

Is the review written in accessible language?

Yes

Are all factual statements correct and adequately supported by citations?

Yes

Are the conclusions drawn appropriate in the context of the current research literature?

Yes

Is the topic of the review discussed comprehensively in the context of the current literature?

Yes

Reviewer Expertise:

Biostatistics, Environmental Statistics, Air pollution Epidemiology, Climate and Health

I confirm that I have read this submission and believe that I have an appropriate level of expertise to confirm that it is of an acceptable scientific standard, however I have significant reservations, as outlined above.

## References

[1] : A Comparison of Three Holistic Approaches to Health: One Health, EcoHealth, and Planetary Health. *Front Vet Sci* .2017;4: 10.3389/fvets.2017.00163 163 10.3389/fvets.2017.00163 29085825 PMC5649127

# Response

**Published:** 2024-10-17

**Authors:** Laurence Mabile

**Affiliations:** CERPOP, Universite Toulouse III Paul Sabatier, Toulouse, Occitanie, France

**Reviewer Comments:**

 Thank you for the opportunity to provide comments on this review article which is a call to action on better integrating the social inequities and societal factors into climate change and health research & policy response. The authors conducted a review of the literature at the intersection of climate change, holistic frameworks of health and the social environment, summarizing trends (and identifying gaps?) with the process diagram in Figure 1.

 The authors then structure this information in the synthetic knowledge framework in Figure 2, and provide a summary of barriers to progress in terms of data-related challenges (Box 1) and recommendations for various stakeholders (Box 2).

 The authors are to be commended for tackling such a large and tricky problem of how to advance the study of climate and health by integrating the social environment. This review will help drive progress, though I do have several comments which may help strengthen the contribution.

***Author Response: *** Many thanks for your attentive reading and for your constructive comments and suggestions.

**Reviewer Comments:**

**Major comments:** It is challenging to operationalize a holistic approach in empirical studies, as shown by only 20% of the reviewed articles being empirical. The general recommendations for researchers in Box 2 are helpful. But it might drive the field forward more quickly to highlight several empirical research case studies (e.g., drawn from the 20%), perhaps sharing strengths/limitations.

***Author Response:*** We have provided, as a supplementary box, three examples of empirical studies that illustrate how integrating social conditions as part of the a holistic view can help better understanding complex human-environmental risks leading to health issues.

 This box (Box A) is now added to the supplementary material of the manuscript at the OSF repository [https://osf.io/6t3j7] and is referred to in the main text at the end of third paragraph of the discussion section.

***Further comment: *** Generally speaking with regards to many infectious disease models, data used in such models imply that all people face and pose uniform risks of infection. Knowledge about socially differentiated exposure could be more integrated into disease modelling efforts to improve the understanding of the heterogeneous dynamics of transmission.

 Social data could also convey more specific and quantitative information if treated as fundamental causes in statistical analyses (i.e. contributive part for each social exposure in a causal inference model leading to health issues through contamination or directly).    

**Reviewer Comments:**

 The recommendations of Neufcourt et al are mentioned twice, but not described. Please add a more context here for the reader.

***Author Response: *** We have now added the following sentence at the end of the Discussion section headed ‘Climate change, social environment & health within holistic frameworks: insights from the literature analysis’:  « A set of methodological recommendations provided by Neufcourt et al. ^18^ can help extend research in this direction.

 These are meant to be used when designing empirical studies aimed at investigating relations between exposome and health outcomes. They highlight questions to ask to make the hypothesised relationships more explicit, especially when testing hypotheses: which approach to use (agnostic / hypothesis driven / mixed) ; when selecting data, which type of social variables to account for (behavioural / material / psychosocial) ; how to treat social factors in analyses ; what are the corresponding limitations. »  

**Reviewer Comments:**

 Epidemiological studies are increasingly using causal thinking and directed acyclic graphs (DAGs), can the authors provide an example of how to operationalize Fig 1&2 in an epi study DAG?

***Author Response:*** Directed acyclic graphs are causal diagrams where statistical interactions between exposures cannot be represented such as the interaction between climate and social conditions. The climate/social/health pattern can however be illustrated conceptually specifying the exposures, the various variable effects and their direction, and interactions. Where we provide a specific example (see Graph A at : https://osf.io/5dc8e] On one side, global urban policies (macro-level of environment) determine people ‘s housing conditions.

 Housing (micro-level) in turn can increase mortality through the quality of the building which may lead to indoor air pollution or mould spore exposure.

 On the other side, nature macro-governance such as human activities impacting GHG emission (e.g. deforestation) contributes to increasing global temperatures. High temperature in turn triggers mortality through hyperthermia.

 Meanwhile, housing can be affected by high temperature depending on various characteristics (insulating capacities, surrounding vegetal cover…). Interaction between both exposures (high temperature x housing) modifies the effect of temperature on increasing mortality.

 Further, human migration (meso-level) affects conditions of housing and worsens mortality. Graph A is referred to, as a supplementary file, in the main text at the end of the Discussion section named *Framing and structuring the complexity.*  

**Reviewer Comments:**

 As described meso-level processes seem well-suited to ecological analysis of administrative data, might be worthwhile to describe this more?  

***Author Response:*** Many thanks for pointing this out. Indeed, while working with administrative data we are more likely to have access to meso and micro data. Analysing macro exposures is rather relevant for inter-country comparisons. We have now specified this in the text (at the end of the paragraph named *Framing and structuring the complexity)*.  

**Reviewer Comments:**

 Fig 1 – I was surprised to not see wildfires or air pollution in the climate clouds.

***Author Response:*** Figure 1 provides a synthesis of the 80 empirical papers identified from our literature review on the basis of the keywords string that did not specifically include the term ‘wildfires’ as they were considered as consequences of climate change.

 Further, each box of Figure 1 (all except purple ones) specifies the number of articles mentioning the social element identified. Wildfires and pollution did not come out from the analysis in any of the 80 articles.

 However, as part of the theoretical pathways that were specified as purple hexagon boxes in Fig.1 -the most likely to link the social components to health outcomes (based on the literature)-, pollution was implicitly considered as ‘food & water safety/quality’.

 The following sentence has now been added in the discussion section of the manuscript : ‘It can be noted that ‘pollution’ did not emerge from the analysis. (at the end of second paragraph of section named *Visualizing the challenging entanglement of social components in climate & health*  

**Reviewer Comments:**

**Minor comments:**

 Introduction, Paragraph 6, Sentence 1: consider citing a review that compares the 3 holistic frameworks of health mentioned, perhaps: Lerner H, Berg C, 2017 [Ref-1]

***Author Response: *** We agree this reference describes well the commonalities and differences between the three approaches. It has now been added where mentioned.

**Reviewer Comments:**

 Discussion paragraph 1. Reconsider use of the word “significant” since readers may think this refers to statistical significance of a hypothesis test

***Author Response: *** The word «significant » has now been replaced by « noteworthy».

**Reviewer Comments:**

 Fig 1/text – English language - consider using “precipitation” rather than “precipitations”

***Author Response: *** The ‘s’ has now been removed in Figure 1.

**Reviewer Comments:**

 Fig 2 – note: with current color choice some words become illegible when printed in black and white.

***Author Response: *** We are aware and sorry for this. We have not found any other ways to integrate all dimensions than using many colors…
